# Neuroplasticity Changes on Human Motor Cortex Induced by Acupuncture Therapy: A Preliminary Study

**DOI:** 10.1155/2017/4716792

**Published:** 2017-02-15

**Authors:** Yi Yang, Ines Eisner, Siqi Chen, Shaosong Wang, Fan Zhang, Linpeng Wang

**Affiliations:** ^1^Acupuncture and Moxibustion Department, Beijing Hospital of Traditional Chinese Medicine, Capital Medical University, Meishuguanhoujie No. 23, Dongcheng District, Beijing 100010, China; ^2^Department of Chinese Medicine, Capital Medical University, Youanmenwai Xitoutiao No. 10, Fengtai District, Beijing 100069, China; ^3^Acupuncture Department, Beijing University of Chinese Medicine, Beisanhuan Donglu No. 11, Chaoyang District, Beijing 100029, China

## Abstract

While neuroplasticity changes measured by transcranial magnetic stimulation have been proved to be highly correlated to motor recovery and have been tested in various forms of interventions, it has not been applied to investigate the neurophysiologic mechanism of acupuncture therapy. The aim of this study is to investigate neuroplasticity changes induced by a single session of acupuncture therapy in healthy adults, regarding the excitability change on bilateral primary motor cortex and interhemispheric inhibition. Ten subjects took a 30-minute acupuncture therapy and the same length relaxing phase in separate days. Transcranial magnetic stimulation measures, including resting motor threshold, amplitudes of motor-evoked potential, and interhemispheric inhibition, were assessed before and 10 minutes after intervention. Acupuncture treatment showed significant changes on potential amplitude from both ipsilateral and contralateral hemispheres to acupuncture compared to baseline. Also, interhemispheric inhibition from the contralateral motor cortex to the opposite showed a significant decline. The results indicated that corticomotoneuronal excitability and interhemispheric competition could be modulated by acupuncture therapy on healthy subjects. The following question about whether these changes will be observed in the same way on stroke patients and whether they correlate with the therapeutic effect on movement need to be answered by following studies. This trial is registered with ISRCTN13074245.

## 1. Introduction

Motor functional recovery after stroke depends on a number of neuroplastic underpinnings. By using transcranial magnetic stimulation (TMS), studies targeting cortical and corticospinal physiology of both the affected and the spared hemisphere have provided us insight into these underlying mechanisms of motor deficits and beneficial effects of therapeutic interventions. One fundamental finding is that patients after stroke exhibit overactive in their contralesional primary motor cortex (M1) and therefore show high level of neural excitability, while the ipsilesional M1 exhibit low excitatory level [1]. The downregulating excitability in the contralesional M1 as well as the upregulating excitability in the ipsilesional M1 are correlated with better motor outcome in stroke patients [2–5]. Another important finding is that the rebalance of interhemispheric competition could play an important role in the process of motor recovery. It is suggested that suppressing the excitability of the unaffected hemisphere could enhance motor recovery by reducing abnormal interhemispheric inhibition (IHI) of the hemisphere affect by stroke [6–8]. Although TMS measures have been used to dissect the mechanism of various forms of treatments [9, 10], it has not been applied to investigate the neurophysiologic mechanism of acupuncture therapy.

Acupuncture is a promising adjuvant intervention that was introduced for the rehabilitation of patients with hemiparesis for decades. It has been applied to stroke patients with motor deficits and led to a remarkable motor recovery [11, 12]. This phenomenon led to studies demonstrating and characterizing physiological mechanisms associated with acupuncture. Previous data on physiological effects of acupuncture assessed by TMS generally suggested an inhibitory effect of specific single acupoint on motor cortex excitability [13–16]. However, it is not yet known that in what way could the clinical acupuncture regimen, comprising multiple acupoints as in the real-world settings, modulate plastic changes in the human primary motor cortex. Moreover, the TMS measure to interhemispheric inhibition (IHI) has not been reported in any previous acupuncture studies. To address these issues, this present exploratory study was conducted. We used TMS to investigate how bilateral M1 excitability and IHI could be modulated by one single session of acupuncture therapy on healthy subject.

## 2. Method

### 2.1. Subjects

Ten healthy volunteers (five males, five females; 24–40 years old, 28.3 ± 5.5 years, mean ± SD) participated in the study. None of the participants had neurological, psychiatric, or other medical problems or reported any contraindication to TMS [17]. All subjects were right-handed according to the Oldfield handedness inventory[18]. The protocol for this study was approved by the Research Ethical Committee of Beijing Traditional Chinese Medicine Hospital (China) and was conducted according to the Declaration of Helsinki. All subjects gave written informed consent before their participation.

### 2.2. Intervention

All subjects underwent both needling intervention (a 30-min period of acupuncture treatment) and control period (a 30-min idle time, with no stimulation) in different days in a randomized order, with an interval of 7 days.

An experienced acupuncturist performed the acupuncture needling with disposable acupuncture needles (Huatuo brand, Suzhou Medical Appliance Factory, Suzhou, China; 0.25 mm diameter, 30 mm length). The acupoints used in this study were based on the acupuncture prescription for the treatment of poststroke motor dysfunction, named “Wang's Extremities' Acupoints Recipe” (*Wang Shi Shou Zu Shi Er Zhen*). The ten acupoints (*Quchi* (LI-11),* Shousanli* (LI-10),* Waiguan* (TB-5),* Hegu* (LI-4),* Zusanli *(ST-36),* Yanglingquan* (GB-34),* Sanyinjiao* (SP-6), and the last three points of* Baxie* (EX-UE9)) used were located on the left forearm, hand and lower leg. The needling methods of “lifting and thrusting” (120 times per minute) and “rotating” (180 degrees, 120 circle per minute) were conducted on each point until the sensation of* Deqi* (a characteristic sensation of aching and tingling) was reported by the subjects. Then, the needles were kept in situ without further stimulation. The control condition was conducted because attentional and cognitive factors vary during the course of the experiment and that could influence cortical excitability [19]. Subjects sat comfortably on an armchair and were instructed to keep relaxed but alert during both the control period and acupuncture intervention.

### 2.3. Transcranial Magnetic Stimulation Measures

Transcranial magnetic stimulation was used to evaluate the corticomotor excitability in each hemisphere as well as interhemispheric inhibition (IHI), before and at 10 min after the experimental intervention (needling/control). The TMS procedure was performed with the subject seated comfortably in a quiet, semidarkened room. The upper limbs were kept supported with muscles in resting condition. Surface electromyogram (EMG) was recorded from the left and right first dorsal interosseous (FDI) muscles with 9 mm diameter Ag/AgCl surface electrodes on a belly tendon montage. Responses were input to an amplifier through filters set at 100 Hz and 3 kHz. They were then digitized at 10 kHz and stored in a computer for later offline analyses (Cambridge Electronics Design, Cambridge, UK).

TMS was delivered to the hand area of the motor cortex on the left (M1_L_) and right hemisphere (M1_R_) using the Magstim 200 stimulator (Magstim Co. Ltd., Whitland, Dyfed, UK), via a figure-of-eight coil (wing diameter 9 cm) oriented to induce current flow in a posterior to anterior direction in the underlying tissues. The coil was positioned over scalp at the “hot spot” for the FDI muscle.

#### 2.3.1. Measures of Corticomotoneuronal Excitability

The resting motor threshold (rMT) and MEP amplitudes were determined, respectively, for each side of FDIs, to elucidate the basic properties of acupuncture-induced plasticity. The rMT was defined as the lowest stimulation intensity that generated motor-evoked potentials (MEPs) ≥ 50 *μ*V amplitude (peak to peak) in the relaxed FDI in at least 5 of 10 consecutive stimuli [20]. Mean MEP amplitudes were obtained in response to 15 TMS stimuli delivered at each of five stimulus intensities: 90, 100, 110, 130 and 150% of the rMT in randomized order at 5 s interstimulus interval. Based on these data, the recruitment curve was constructed to relate the amplitude of the response to the TMS stimulation intensity. Cortical stimulation was consistently performed first on the left hemisphere followed by the right hemisphere.

#### 2.3.2. Interhemispheric Inhibition

Interhemispheric inhibition effect from the right hemisphere to the left hemisphere was evaluated by delivering paired-pulse stimuli bilaterally (Bistim module, Magstim Co. Ltd.) to the previously determined M1 hotspot for FDIs while participants maintained the muscles in a full relaxed position. The testing stimulus (TS) was set at intensity of +30% rMT in the right FDI muscle. The conditioning stimulus (CS) intensities were randomized at intensities +0%, +10%, +30%, and +50% rMT of the left FDI. Recording blocks consisted of 15 MEPs, the interstimulus interval between CS and TS was set as 10 ms, and the interval between each paired stimuli was 5 s. The amplitude of the conditioned response from IHI was expressed as a percentage of the size of mean MEP amplitude of test stimuli alone.

### 2.4. F-Wave

Supramaximal electrical stimulation was performed in five subjects in a separate session, to measure the F-wave from the left FDI. This measure was chosen to test the effect of acupuncture therapy at the spinal level and therefore to differentiate excitability changes at cortical or subcortical sites [21]. F-waves were recorded and averaged before and 10 min after acupuncture intervention.

## 3. Statistical Analysis

SPSS software (version 23.0, SPSS Inc., Chicago, IL) was used for statistical analysis. Two-way repeated-measures ANOVA was used to compare rMTs over treatments on the same side of FDI before and after the intervention (factors: TIME and TREATMENT). Differences of intervention-induced changes on MEP amplitudes from the recruitment curve were compared with three-way repeated-measures ANOVA model with factors “TIME” (pre- and postintervention), “TREATMENT” (acupuncture and control), and “INTENSITY” (five levels). Bonferroni's post hoc test was used for further analyses. Changes of IHI were compared with three-way repeated-measures ANOVA model with factors “TIME” (pre- and postintervention), “TREATMENT” (acupuncture and control), and “INTENSITY” (four levels). The significance level was set at *P* < 0.05. Unless otherwise stated, values are reported as mean ± standard deviation. All ANOVA results are given with the *F* value and *P* value.

## 4. Results

Based on the acupuncture prescription for poststroke motor dysfunctional treatment, acupuncture needling for this experiment was performed on the left arm and leg. All TMS assessments were performed on both hemispheres, monitoring EMG on left and right FDI muscles. All thresholds are expressed as a percentage of maximum stimulator output (%MSO).

### 4.1. Effects of Acupuncture on rMT

**Figure 1:**
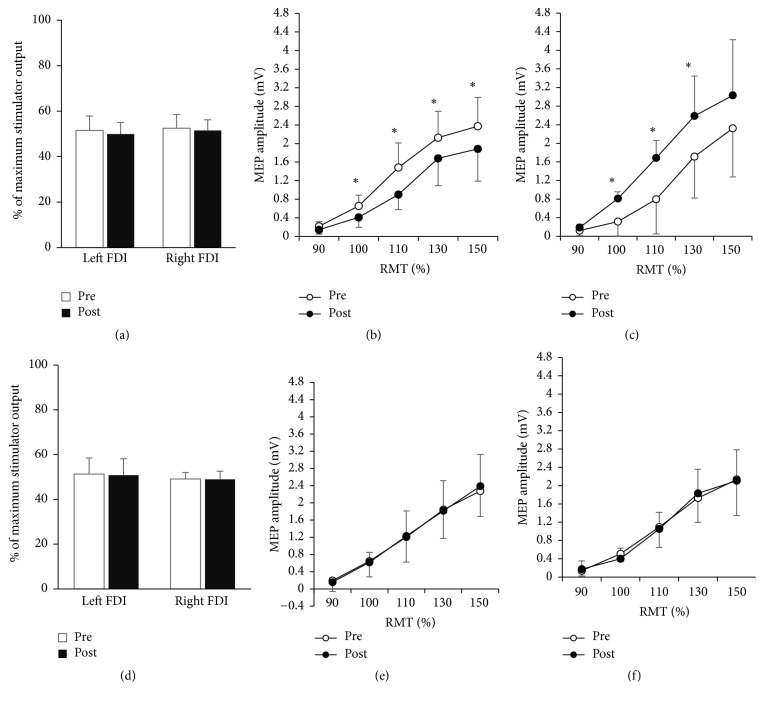
TMS properties of intervention-induced plasticity. (a–c) Effects of acupuncture on TMS variables. (a) Motor thresholds (mean ± SD). Acupuncture affected the RMT of neither the contralateral nor ipsilateral FDI of acupuncture (*P* > 0.5). Preintervention (open bars); postintervention (filled bars). (b) The recruitment curve (mean ± SEM) from the left FDI (ipsilateral FDI of acupuncture). The ordinate gives the MEP size in mV; the abscissa shows the stimulus intensity relative to RMT. Preintervention (○); postintervention (●). Significantly smaller MEPs were elicited at intensities greater than 90% RMT after acupuncture. (c) The recruitment curve (mean ± SEM) from the right FDI (contralateral FDI of acupuncture). Significantly larger MEPs were elicited at intensities 100%, 110%, and 130% RMT after acupuncture. (d–f) Variables by the control intervention. (d) Motor thresholds (mean ± SD). RMT in neither the left FDI nor the right FDI was altered by the control period. (e) The recruitment curve (mean ± SEM) from the left FDI. No significant changes of MEP amplitude were elicited by the control period. (f) The recruitment curve (mean ± SEM) from the right FDI. No significant changes of MEP amplitude were elicited by the control period. ^*∗*^*P* < 0.01 (Bonferroni's post hoc adjustment).

Neither the acupuncture intervention nor the relaxing period altered rMT (Table 1). Mean rMTs for the left FDI were 51.50 ± 6.364 preintervention and 49.90 ± 5.109 postintervention for the acupuncture treatment, and 51.30 ± 7.17 preintervention and 50.80 ± 7.361, postintervention for the control condition. Mean rMT for the right FDI were 52.50 ± 5.986 preintervention and 51.50 ± 4.720 postintervention for the acupuncture treatment and 49.10 ± 2.923 preintervention and 48.90 ± 3.695 postintervention for the control. Two-way repeated-measures ANOVA showed no significant effects of intervention nor time or on either left FDIs (TREATMENT,* F *= 0.016, *P* = 0.902; TIME,* F *= 2.702, *P* = 0.135; TREATMENT × TIME interaction,* F *= 0.595, *P* = 0.460) or right FDIs (TREATMENT,* F *= 2.201, *P* = 0.172; TIME,* F *= 0.764, *P* = 0.405; TREATMENT × TIME interaction,* F *= 0.310, *P* = 0.591) (Figures 1(a) and 1(d)).

### 4.2. Effects of Acupuncture on MEP Amplitude

According to the three-way ANOVA, the pattern of MEP amplitudes differed significantly between the acupuncture and control intervention across time for both sides of FDIs, respectively (Table 1). For the left FDI, significant effects were shown on TREATMENT (*F *= 6.202, *P* = 0.034), TIME × TREATMENT interaction (*F *= 19.431, *P* = 0.002) and TIME × TREATMENT × INTENSITY interaction (*F *= 6.049, *P* = 0.027). This indicates that the two interventions modulated MEP in significantly different ways across time. Similarly, for the right FDI, it also showed significant effects of TREATMENT (*F *= 7.151, *P* = 0.025), TIME × TREATMENT interactions (*F *= 25.475, *P* = 0.001), and TIME × TREATMENT × INTENSITY interactions (*F *= 4.558, *P* = 0.049).

Post hoc analysis using Bonferroni's adjustment revealed that the MEP amplitudes were modulated only by acupuncture, on both left and right FDI, but not the control period. There were significantly smaller MEPs on the left FDI elicited at all the four intensities above 90% RMT after acupuncture than baseline (Figure 1(b) and Table 2). Meanwhile, the right FDI showed significantly larger MEPs elicited at intensities 100%, 110% and 130% rMT after acupuncture than baseline (Figure 1(c) and Table 2). On the contrary, the control intervention induced no significant changes on neither sides of FDI across time (*P* > 0.05 for both sides at all intensity levels, Figures 1(e) and 1(f)).

### 4.3. Effects of Acupuncture on IHI

**Figure 2:**
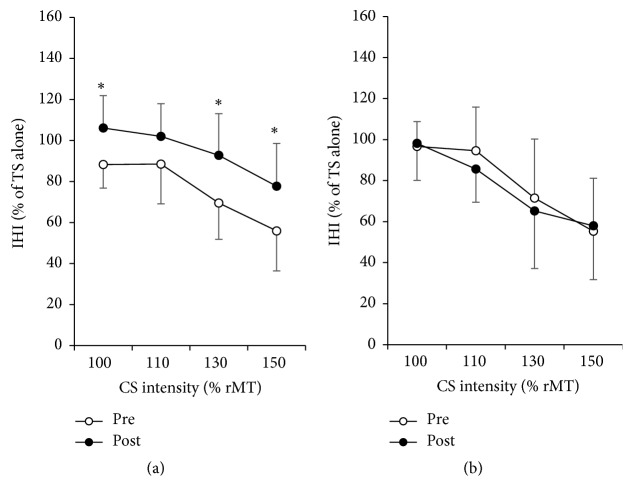
Interhemispheric inhibition (IHI) curves. IHI curves (*N* = 10) show inhibition effect from the M1 in the right hemisphere (where the conditioning stimulus (CS) was delivered) to the M1 in the left hemisphere (where the testing stimulus (TS) was delivered). The abscissa indicates the CS intensities expressed as a fraction of rMT. The ordinate indicates the amplitude of conditioned MEP from the left M1 expressed as a percentage of the MEPs from TS alone. Higher values represent lower inhibition from the right M1 to the left M1. Preintervention (○); postintervention (●). After the intervention, significantly lower inhibitions from CS to TS were observed from the treatment of acupuncture (a) but not the control (b). ^*∗*^*P* < 0.0125 (Bonferroni's post hoc adjustment).

The three-way ANOVA analysis showed that the percentage of the size of MEP amplitudes from TS performed differently between the two interventions across time points (TIME × TREATMENT interaction, *F* = 11.976, *P* = 0.007) (Table 1). Post hoc analysis with Bonferroni's adjustment showed significant increment after the acupuncture treatment on the percentage of MEP amplitudes from TS at CS intensity 100%, 130% and 150%, which indicated a declined inhibition from the right M1 (contralateral to acupuncture side) to the left M1 (ipsilateral to acupuncture side) (Figure 2(a) and Table 2). In contrast, no significant difference was shown after the control condition (Figure 2(b)).

### 4.4. Site of Changes in Motor Excitability (F-Wave)

The mean F-wave amplitudes in the left FDI before and after acupuncture intervention were not significantly different at 198.00 ± 25.48 *μ*V and 218.20 ± 36.27 *μ*V, respectively (*t* = 1.822, *P* = 0.143).

## 5. Discussion

This is the first study that investigated the neuroplastic mechanism of acupuncture therapy on cortical excitability and interhemispheric inhibition. The acupuncture therapy was found to induce a significant modulation of MEP amplitudes of the motor pathways that depart from the contralateral primary motor cortex. Changes in MEP amplitude also occur on the ipsilateral corticospinal excitability following acupuncture. No significant changes of peripheral excitability (F-wave) were observed before and after acupuncture. The results indicate that changes in corticomotoneuronal excitability could be elicited by acupuncture intervention. Also, the interhemispheric inhibition from the contralateral M1 to the ipsilateral M1 showed a significant decline, which might explain the change on ipsilateral M1.

The recruitment curve of MEP amplitudes is a convincible parameter for cortical excitement level [10]. The decrease of the recruitment curve on the hemisphere contralateral to acupuncture suggested a reduction in the excitability level of the contralateral M1. The results of F-wave further ruled out the possibility of excitability change of the motoneuronal pool in the spinal cord, which has been previously addressed in other studies and gave evidence to the supraspinal mechanism for this acupuncture-induced plasticity change [13, 14].

The rMT and MEP amplitudes were chosen in this study as measures of neuroplasticity change. While there were noticeable changes on MEP amplitudes, few modulations were altered on rMT after acupuncture. Although both rMT and MEP amplitude are measures of cortical excitability, rMT is mainly affected by mechanisms of neuronal membrane excitability involving sodium and calcium channels [22, 23]. In contrast, MEP amplitudes are predominantly influenced by changes in synaptic excitability, which was evidenced by alterations in presence of pharmacological modifiers for synaptic transmission [24]. Our results imply that acupuncture might modulate the cortical excitability by influencing the activity of neural synapsis, instead of the neuronal membrane.

Our results show that acupuncture also influenced the excitability of the ipsilateral hemisphere. The increasement of the MEP amplitudes on the hemisphere ipsilateral to acupuncture indicate an increase of excitability of the ipsilateral M1. A reasonable explanation of this excitability increment is the transcallosal pathway model. As mentioned above, the acupuncture intervention reduced the excitability level of the contralateral M1. As a result, its inhibitive influence toward the opposite site–the ipsilateral M1, might also be declined. This assumption was supported by the change of IHI curves, which showed that the inhibitory effect from the right M1 (contralateral to acupuncture side) to the left M1 (ipsilateral to acupuncture side) declined significantly after acupuncture intervention. Since the ipsilateral M1 received less inhibition, its excitability increased. It might be the explanation of how acupuncture modulates the excitability of the ipsilateral M1. Besides, since acupuncture could modulate the interhemispheric activity, which has been proved to be an important mechanism underlying motor recovery, this model could be one possible explanation of the effect of acupuncture treatment for poststroke dysfunctions.

According to Chinese medical theory, acupuncture therapy functions through the regulation of* Qi* (the energy). There are twelve main channels and meridians (called* Jing Luo*) that located all over the body and connected in circles with certain orders. The* Qi* keeps running through the twelve channels and meridians and this takes it 28 minutes to finish one whole round. Therefore, the therapeutic effect of stimuli on acupoints could travel to everywhere around the body through the channels and meridians, carried by the circular movement of* Qi*. The needling in the left extremities could then modulate not only the body function nearby the acupoints but also the function of the distant organs, including the ipsilateral and contralateral hemisphere.

In this study, the acupuncture intervention with acupoint formula for the treatment of motor recovery after stroke is demonstrated being capable of modulating the excitability of primary motor cortex as well as the interhemispheric competition, which have been proposed as essential mechanisms for motor recovery on stroke patients. However, results from this study could not answer the question how this therapy could affect the neuroplasticity on poststroke brain. After a stroke attack, the human brain shows various changes based on the location and range of the lesion. Therefore, future studies should be conducted using acupuncture treatment on patients sorted with lesion location (cortical, subcortical, etc.) to figure out the effect of acupuncture therapy on poststroke neuroplasticity.

There are other sorts of technique that can modulate cortical excitability and have been explored as a possible adjuvant of poststroke neurorehabilitative treatment. For example, previous studies have suggested that noninvasive brain stimulation (NIBS), including repetitive transcranial magnetic stimulation (rTMS) and direct current stimulation (tDCS), may be beneficial to motor recovery after neurological injury, by modulating cortical motor excitability from both the ipsilesional and contralesional hemispheres [3, 25–27]. Meanwhile, it has been proven that the plasticity of primary sensorimotor cortex is also responsive to peripheral sensory stimulations. Besides acupuncture, peripheral electrical nerve stimulation, as a form of simple, painless somatosensory input, has been demonstrated to modulate corticomotoneuronal excitability [28]. The mechanisms underlying this sensorimotor cortical plasticity are still not well understood. One possible mechanism involves the thalamic nucleus, which receives somatosensory input while links to the primary motor cortex by direct projections. Another hypothesis is that the site of this sensory-motor interaction located at the motor cortex itself [29]. It is also possible that other motor cortical areas which receive somatotopically organized somatosensory information are involved in this sensorimotor interaction [28]. As a unique kind of peripheral sensory stimulation, acupuncture may share some of these mechanisms with regard to its effectiveness on cortical plasticity. However, since it has quite different characteristics from peripheral electrical stimulation (e.g., acupuncture induces pain during treatment), its mechanism requests further research.

The main limitation of this study is the small sample size. Also, as for the method of control, no sham acupuncture was applied in the present study. According to the Standards for Reporting Interventions in Clinical Trials of Acupuncture (STRICTA), sham acupuncture is a proper control method for acupuncture studies. There are commonly recommended methods as sham acupuncture intervention, such as needling unrelated or sham acupoints and superficial needling. However, as a preliminary study, the aim of this research is to demonstrate the effect of acupuncture treatment on cortical activity, instead of comparing the effect of different acupoints or needling techniques. Hence, idle period, as a classic design for control in cortical excitability research, rather than sham acupuncture, was identified as the optimal method for the control design in this study.

## 6. Conclusion

Our finding indicated that acupuncture could modulate the excitability of M1 as well as the interhemispheric inhibition on healthy subjects. The following question about whether these changes will be observed in the same way on stroke patients and if it is correlated with the beneficial effect on behavioural improvement need to be answered by following studies.

## Figures and Tables

**Table 1:** General comparisons by two-way/three-way repeated-measures ANOVA on TMS measures of cortical excitability from both hemisphere and IHI.

	df	*F* value	*P* value
*RMT*			
*Left FDI*			
TIME	1,9	2.702	0.135
TREATMENT	1,9	0.016	0.902
TIME *∗* TREATMENT	1,9	0.595	0.460
*Right FDI*			
TIME	1,9	0.764	0.405
TREATMENT	1,9	2.201	0.172
TIME *∗* TREATMENT	1,9	0.310	0.591
*MEP amplitudes*			
*Left FDI *(*n* = 10)			
TIME	1,9	29.474	<0.001^*∗*^
TREATMENT	1,9	6.202	0.034^*∗*^
INTENSITY	4,6	44.853	<0.001^*∗*^
*TIME ∗ TREATMENT*	1,9	19.431	0.002^*∗*^
TIME *∗* INTENSITY	4,6	1.930	0.225
TREATMENT *∗* INTENSITY	4,6	2.418	0.160
TIME *∗* TREATMENT *∗* INTENSITY	4,6	6.049	0.027^*∗*^
*Right FDI *(*n* = 10)			
TIME	1,9	48.285	<0.001^*∗*^
TREATMENT	1,9	7.151	0.025^*∗*^
INTENSITY	4,6	27.591	0.001^*∗*^
*TIME ∗ TREATMENT*	1,9	25.475	0.001^*∗*^
TIME *∗* INTENSITY	4,6	4,722	0.046^*∗*^
TREATMENT *∗* INTENSITY	4,6	8.096	0.013^*∗*^
TIME *∗* TREATMENT *∗* INTENSITY	4,6	4.558	0.049^*∗*^
*IHI*			
TIME	1,9	2,739	0.132
TREATMENT	1,9	3.480	0.095
INTENSITY	3,7	16.012	0.002^*∗*^
*TIME ∗ TREATMENT*	1,9	11.976	0.007^*∗*^
TIME *∗* INTENSITY	3,7	1.425	0.314
TREATMENT *∗* INTENSITY	3,7	0.879	0.496
TIME *∗* TREATMENT *∗* INTENSITY	3,7	1.128	0.401

RMT: resting motor threshold; MEP: motor-evoked potential; IHI: interhemispheric inhibition; FDI: first dorsal interosseous; df: degrees of freedom. ^*∗*^*P* < 0.05.

**Table 2:** Post hoc comparison by paired *t*-test on MEP amplitudes and IHI before and after acupuncture.

	Time 1	Time 2	*t* value	*P* value
*MEP amplitudes (mV)*				
*Left FDI*				
90% RMT	0.218 ± 0.099	0.137 ± 0.091	2.616	0.028
100% RMT	0.658 ± 0.229	0.405 ± 0.221	3.333	0.009^*∗*^
110% RMT	1.485 ± 0.526	0.837 ± 0.359	4.407	0.002^*∗*^
130% RMT	2.129 ± 0.565	1.509 ± 0.494	4.159	0.002^*∗*^
150% RMT	2.372 ± 0.620	1.732 ± 0.666	4.738	0.001^*∗*^
*Right FDI*				
90% RMT	0.124 ± 0.069	0.189 ± 0.108	−1.648	0.134
100% RMT	0.317 ± 0.145	0.812 ± 0.309	−4.169	0.002^*∗*^
110% RMT	0.796 ± 0.376	1.683 ± 0.744	−3.781	0.004^*∗*^
130% RMT	1.714 ± 0.859	2.587 ± 0.892	−3.769	0.004^*∗*^
150% RMT	2.323 ± 1.198	3.031 ± 1.046	−2.859	0.019
*IHI (%)*				
100% RMT	88.281 ± 15.831	106.079 ± 11.508	−3.556	0.006^*∗*^
110% RMT	88.497 ± 15.868	102.046 ± 19.423	−1.945	0.084
130% RMT	69.545 ± 20.314	92.743 ± 17.763	−3.826	0.004^*∗*^
150% RMT	55.937 ± 20.865	77.705 ± 19.524	−4.410	0.002^*∗*^

MEP: motor-evoked potential; IHI: interhemispheric inhibition; FDI: first dorsal interosseous.

Time 1. Before acupuncture intervention.

Time 2. 10 min after the removal of all needles.

^*∗*^ *P* < 0.01 for MEP analysis and *P* < 0.0125 for IHI, as Bonferroni's post hoc adjusted significant level.
